# Supplementary material for: The association between weight at birth and breast cancer risk revisited using Mendelian randomisation
Source: Eur J Epidemiol. 2019 Feb 8;34(6):591–600. doi: 10.1007/s10654-019-00485-7 (PMC6497616; doi:10.1007/s10654-019-00485-7)
Supplement: Supplementary file 1 — Supplementary material 1 (DOCX 334 kb) [file 10654_2019_485_MOESM1_ESM.docx]

**Supplementary Figure 1.**

(a)
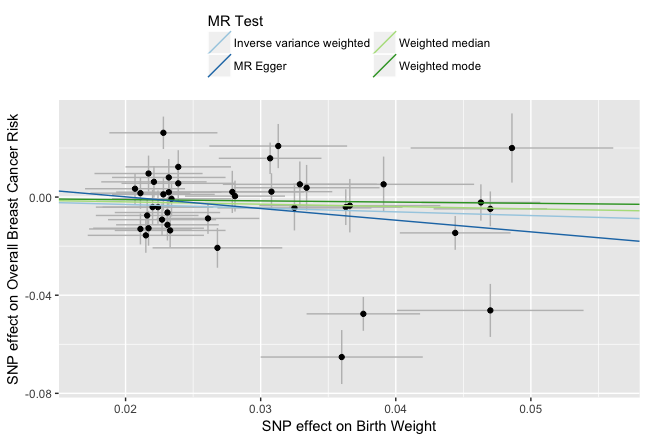
(b)
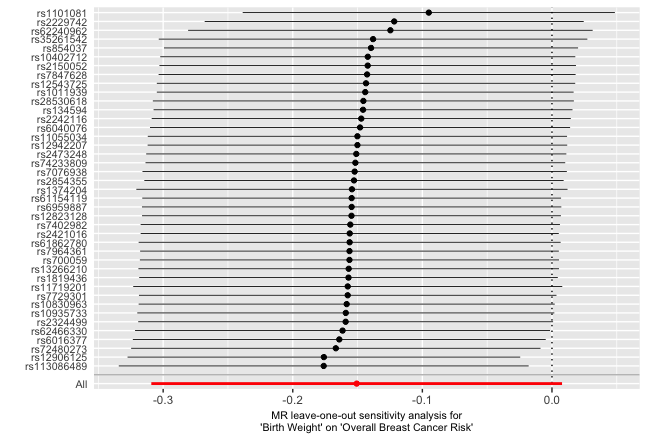


(c)
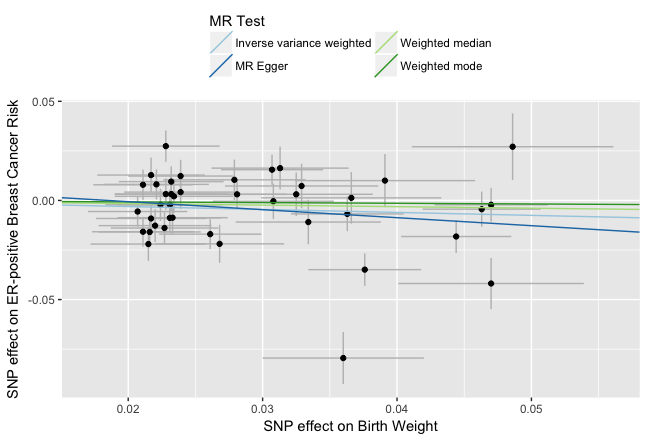
(d)
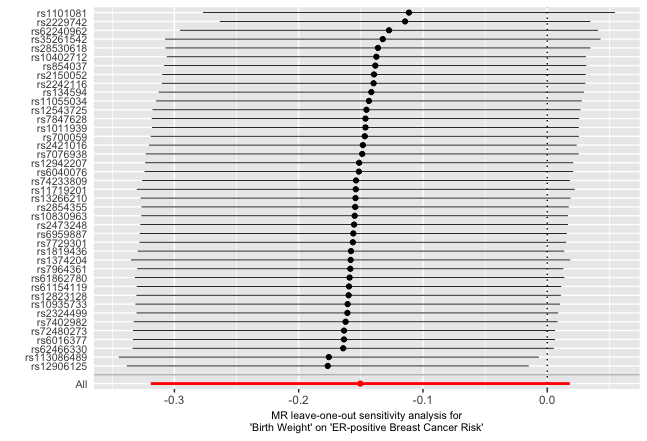


(e)
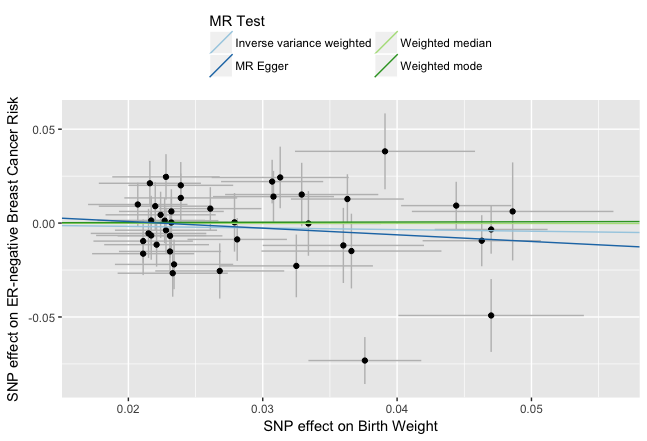
(f)
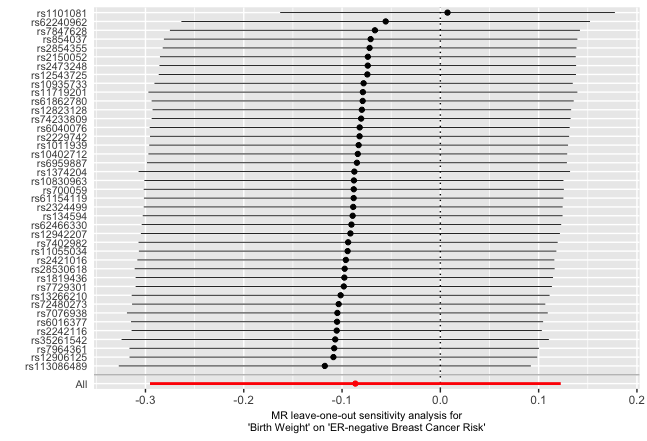


**Supplementary Figure 1:** Scatter plots of associations between SNPs and breast cancer against SNP-birth weight associations for **(a)** overall breast cancer, **(c)** estrogen receptor (ER)-positive breast cancer, and **(e)** ER-negative breast cancer with the estimates from the different Mendelian randomisation methods indicated by the coloured lines (see inset legend). Leave-one-out permutation analysis plots for **(b)** overall breast cancer, **(d)** estrogen receptor (ER)-positive breast cancer, and **(f)** ER-negative breast cancer obtained by leaving out the SNP indicated on the X-axis and repeating the standard inverse-variance weighted method with the rest of the 41-SNP instrumental variable used for univariable Mendelian randomisation analysis in this study.
